# The X-Rays

**Published:** 1901-06-15

**Authors:** 


### Hospital Clinics and Medical Progress.

### THE X-RAYS.

On the first discovery of the X-rays, so wonderful did the results appear that people were perhaps to be excused for taking somewhat exaggerated views regarding the information given by skiagraphy. Further experience, while fully confirming all that had been hoped in regard to the utility of the process in the hands of those who have learned by experience how to appraise and interpret the results, has served to show under how many limitations these results must be received. It must be remembered that in the X-rays we are dealing with an agent of the nature of which we know hardly anything, and over the effects of which we have practically no control; that the whole method is a mere matter of empiricism, and that until we know more about it we must be very guarded in our judgment of what they show or fail to show. Mr. Golding-Bird[1] summing up this matter says that "for the present, at least, a practitioner who has not considerable experience of skiagraphy should be very shy of using it to test his own work, or without skilled aid of thinking to successfully rebut a charge brought against him on the strength of a skiagraph produced by a patient." The latter is an important point to which we have referred on previous occasions. "The sooner," says Mr. Golding-Bird, "the public is instructed in the uncertain indications that skiagraphy gives, the sooner will lawyers learn that it is not a reliable witness—less so indeed than ordinary photography—and the less ready will a certain class of them—well known to our profession—be to seize on a skiagraph to 'make a case' and use it as a motive to urge on a weak but greedy client to bring an undeserved charge of malpractice against his doctor."

### X-Rays in Cancer.

While on the subject of the X-rays, we must record as an interesting fact, without at present expressing any opinion as to its deeper meaning, the remarkable results which have recently been produced in a case of cancer of the breast, under the care of Mr. Andrew Clark,[1] in the Middlesex Hospital. The patient was a woman 60 years of age, whose right breast, on admission, consisted almost entirely of a red ulcerating surface with a hard margin, the whole being firmly fixed to the pectoral muscle and bony structures beneath, while under the skin at the edge of the ulceration towards the axilla a hard mass could be felt. In the axilla several enlarged and indurated glands could be distinguished. The disease had probably commenced about the year 1890. In 1894 operation had been advised but refused. On March 16th, 1901, Mr. Clifford, house-surgeon at Middlesex, asked to be allowed to try the effect of the X-rays upon this ulcerated surface, and on the same day this treatment was commenced. It was continued for five weeks, the surface being exposed to the rays five days a week for fifteen minutes at a time. On March 17th the breast was a little cleaner and numerous small islands of epithelium were present. On April 23rd there was still further improvement in the ulceration, and a diminution in the size of the indurated lump. On May 14th it was noted that the induration had gradually been getting less and that the patient had but little pain; the glands in the axilla were smaller and the general condition of the patient was improving. The marked improvement which took place is well shown in the photograph by which Mr. Clark's paper is illustrated. We may note at the same time that at the recent annual meeting of the Association of American Physicians held in Washington, Dr. Francis H. Williams[2] reported a series of cases in which the X-rays had been used with good effect in cases of cancer. It is extremely difficult to formulate any idea as to the manner in which the X-rays can produce such results, but as we know that whatever else the rays may do, they at least go through the tissues, and as we know that in their course they do sometimes produce such an alteration in nutrition as to cause what are spoken of as X-ray burns, our very ignorance as to how these things happen leaves the imagination free to build up all sorts of notions as to the *modus operandi* of the rays in modifying disease processes. It is, however, as yet too early even to guess whether the improvement in such cases as the one here mentioned may be due to a "stimulating" influence exercised by the rays upon the tissues, or to a destructive influence upon some problematical "germ," or even to an interference with nutrition, analogous perhaps with that by which an X-ray burn is produced, but exercised in this case upon the ingrowing epithelium, the invasion of the tissues by which is the essence of the cancer process. Cancer clearly is a case for the application of the Harveian injunction to search out the truth by means of experiment, and happily we seem now to see some hope of the search being not entirely unfruitful.

### Skiagraphy and Fractures.

In dealing with cases of fracture skiagrophy is of use to the surgeon in two ways, as an aid to diagnosis and as a means of judging of results. In by far the larger proportion of cases one is called upon to treat its employment as quite unnecessary, but in a certain class of doubtful cases skiagraphy is of the greatest utility to the surgeon if he constantly bears in mind the limitations under which its results must be accepted. These doubtful cases, says Mr. Golding-Bird, are of two kinds: one in which, although the surgeon is certain of the existence of a fracture, he is in doubt either as to its exact location or its direction, or perhaps its involvement of a joint, and he requires all the information possible for the successful treatment of the case; the other in which the very existence of a fracture is doubtful.

This doubt may not of necessity influence the line of treatment, but if the patient should afterwards get skiagraphed on his own account, and be able to demonstrate a fracture, he might if of a litigious temperament make the position of the surgeon awkward to say the least of it. Among the common fractures those of the fibula, vertical and transverse fractures anywhere, and certain fractures about the hip may prove most difficult of diagnosis except with the aid of the X-rays. Among such difficult cases Mr. Golding-Bird particularises the following: (1) Cases of Pott's fracture. In this injury there may be every degree from the so-called simple sprain up to the thorough-going "Pott," and the deformity may be very slight, never indeed having existed or having been reduced by the "first-aid" manipulations which the case has undergone. (2) Vertical fracture into the knee-joint without deformity. (3) Purely transverse fractures, notably in the lower end of the tibia, for such people can stand, and the condition closely simulates a sprain. (4) Cases of injury to the hip, which occur mostly either among the young or in the distinctly old. In the former a separation of the epiphysis of the head of the femur takes place together, probably, with some splinter torn off the neck. Such a case may run about as usual even for two or three weeks, when the line of separation may yield and the full symptoms of intracapsular fracture may be developed. In old people cases get diagnosed as contused hip in consequence of the tape showing no appreciable shortening and the eversion being but little marked, notwithstanding that a fracture exists which might be demonstrated by the X-rays.

As a Criterion of Progress.

When a surgeon brings his work to the judgment of the rays, he is likely to be shocked to find how imperfect they often appear by the bare and bald evidence of the skiagraph, and until it is recognised that a result which is clinically and practically good may seem abominable from an X-ray point of view, the skiagraph will form an entirely unreliable criterion of success in "setting a fracture." What do we mean by this phrase? When we say that a fracture is well "set" do we mean that the parts are restored to their old position—that the skiagraph of the result shall show merely the slightest possible deviation of outline, and a linear mark across the bone, as well as perfect restoration of the utility of the limb—or do we accept it as sufficient that the function and utility of the limb shall be restored even though the bone scar may be shown by the skiagraph to be far from perfect? No doubt there are many surgeons who will answer that in a "well set" fracture the fragments are in fact restored to their proper places and the broken surfaces placed in contact with each other. All experience until the X-rays came went to show that this was so. Now, however, we know that in innumerable cases, perfect as is the functional result, and good as is the appearance of the limb, the bones are by no means accurately fitted to one another, and the bony scar looks like a great chasm. Mr. Golding-Bird says that in the bulk of fractures "set" and treated in the best possible manner short of operation the skiagraphs show an apparent maladaptation of the bones which, taken alone would seem to indicate a hopeless failure on the part of the surgeon. Yet the patient gets well. We have then to recognise the fact that if the final appeal in every case is to be not the utility of the limb but the beauty of the bone scar, then every case of indirect fracture must be operated on and the bones be adapted by screws or wires, or some other means. Surgeons must then decide how they will advise their patients; "whether to submit to an operation which, from the very circumstances of the case, carries with it more risks of septicism, in spite of the greatest care, than many other operations on bone, in order to obtain a good-looking scar in the bone at the expense of an ugly one in the skin, or whether they will forego all this and be content with a good functional result without operation, but one which will not bear the test of the X-rays as an example of cabinet work." This is no doubt sound teaching, and will be found very useful as a counterblast to what "the patient and his legal adviser . . . think they see in the skiagraph" and to "the conclusion they in their ignorance draw therefrom." To appreciate, however, the full humour of these remarks, it must be remembered that they are made by one of the surgeons to Guy's Hospital, where, in the wards of another surgeon, is the very home of "cabinet work" surgery, with its nails and screws, chisels, mallets, and all the rest of it! In judging of the results of fracture by the X rays one must further bear in mind that a skiagram can in certain position show a fracture where none exists; that in using it it is possible to miss seeing a fracture; that it is possible to show a fracture as still existing, though long united; and that an appearance of distortion can be produced. Clearly, then, neither in diagnosis nor in estimating results can the dicta of the skiagraph be accepted as conclusive by themselves. They are to be made use of by the surgeon as "another aid," and are to to be considered in their proper relation with all the aids we otherwise possess, in judging of the condition of affairs existing in a case of fracture.

[1] Brit. Med. Jour., June 8. [2] New York Med. Rec., May 11.

